# Sugar-based micro/mesoporous hypercross-linked polymers with in situ embedded silver nanoparticles for catalytic reduction

**DOI:** 10.3762/bjoc.13.120

**Published:** 2017-06-22

**Authors:** Qing Yin, Qi Chen, Li-Can Lu, Bao-Hang Han

**Affiliations:** 1 College of Chemistry, Xiangtan University, Xiangtan 411105, China; 2 CAS Key Laboratory of Nanosystem and Hierarchical Fabrication, CAS Center for Excellence in Nanoscience, National Center for Nanoscience and Technology, Beijing 100190, China

**Keywords:** catalytic reduction, hypercross-linking, porous polymers, silver nanoparticles, sugar

## Abstract

Porous hypercross-linked polymers based on perbenzylated monosugars (**SugPOP-1–3**) have been synthesized by Friedel–Crafts reaction using formaldehyde dimethyl acetal as an external cross-linker. Three perbenzylated monosugars with similar chemical structure were used as monomers in order to tune the porosity. These obtained polymers exhibit microporous and mesoporous features. The highest Brunauer–Emmett–Teller specific surface area for the resulting polymers was found to be 1220 m^2^ g^−1^, and the related carbon dioxide storage capacity was found to be 14.4 wt % at 1.0 bar and 273 K. As the prepared porous polymer **SugPOP-1** is based on hemiacetal glucose, Ag nanoparticles (AgNPs) can be successfully incorporated into the polymer by an in situ chemical reduction of freshly prepared Tollens’ reagent. The obtained AgNPs/**SugPOP-1** composite demonstrates good catalytic activity in the reduction of 4-nitrophenol (4-NP) with an activity factor *k*_a_ = 51.4 s^−1^ g^−1^, which is higher than some reported AgNP-containing composite materials.

## Introduction

Hypercross-linked polymers (HCPs) are microporous organic materials with a high specific surface area (SSA) [1–2]. The preparation of HCPs mainly includes three different synthesis strategies, namely postcross-linking of polymeric precursors containing functional groups [3], the “knitting” of rigid aromatic building blocks by external cross-linkers [4], and self-polycondensation of small molecular monomers [5]. Since the Tan group proposed the new synthetic strategy that "knits" low functionality rigid aromatic compounds with formaldehyde dimethyl acetal (FDA) as an external cross-linking agent through a Friedel–Crafts reaction to synthesize a polymer network with a high SSA [6], HCPs with knitted building blocks have been widely utilized because of their high SSA [7–8], mild synthesis conditions [9–10], and wide range of monomers [11].

The porosity and functionality of HCPs is highly dependent on the core structural monomers [12–15]. However, most aromatic skeleton monomers are non-renewable and could generate additional environmental problems. Therefore, the selection and use of low cost, green, raw materials is critical. Sugars are a ubiquitous resource, which plays many different and important roles in the world [16]. The chemical structure of monosaccharides is commonly a polyhydroxylated aldehyde or ketone with a pyranose ring structure. These hydroxy groups can be easily benzylated to afford sugar-based monomers containing multiple aromatic skeletons. Recently, Liu and Dai have reported a class of novel microporous HCPs based on carbohydrates for carbon dioxide capture and storage by hydrogen bonding and dipole–quadrupole interactions [17]. The reported pore-size distribution (PSD) and related porosity tuning are in the range of micropore size. Considering that the polyhydroxylated and chiral structure derived from the monosaccharide has certain effects on the SSA and PSD of the prepared porous polymers, porosity tuning can be likely achieved with varying monomer molecular structures. Moreover, the aldehyde or ketone groups of the material provide the possibility for further modification and functionalization of the materials.

Silver nanoparticles (AgNPs) have received extensive attention because of their unique properties and applications in catalysis [18], antibacterial use [19], phase separation [20], surface-enhanced Raman scattering (SERS) [21], etc. Compared with bulk silver, AgNPs have a more negative reduction potential and higher SSA, which make them more effective in catalytic reactions [22]. However, AgNPs with high surface energy are subject to certain limitations in catalysis due to their extreme tendency to aggregate. In order to solve this problem, an effective method is to encapsulate or embed the AgNPs into a supporting matrix. The loading of AgNPs on different substrates has been reported, for instance, SiO_2_ [23], TiO_2_ [24], Al_2_O_3_ [25], porous carbon [26], carbonaceous matrix [27], carboxymethyl chitosan [28], zeolite [29], cellulose [30], ZnO paper [31] and polymers such as PVP [32–34]. In the catalytic process, porous organic polymers with a high SSA, low framework density and permanent porosity represent a new type of catalyst support [35–37]. The porosity of the matrix can particularly improve the efficiency of the catalyst due to the promotion of the reactant molecules into the holes with the catalyst active sites [38–39]. Therefore, it is of great interest to improve the catalytic efficiency by encapsulating the nanocatalyst in a porous organic polymer.

Keeping these issues in mind, three novel sugar-based porous organic polymers **SugPOP-1–3** were designed and synthesized using a Friedel–Crafts hypercross-linking reaction via knitted perbenzylated monosugars by FDA. Three perbenzylated monosugars **Sug-1–3** having similar chemical structure were used as monomers to tune the porosity and PSD. The SSA values of the obtained porous polymer are around 1000 m^2^ g^−1^. As the porous polymer **SugPOP-1** is based on hemiacetal glucose, it was further postfunctionalized to embed the AgNPs into the material using an in situ chemical reduction of the freshly prepared Tollens’ reagent. The related catalytic reduction by the AgNPs/**SugPOP-1** composite was also explored at room temperature.

## Results and Discussion

**Scheme 1:**
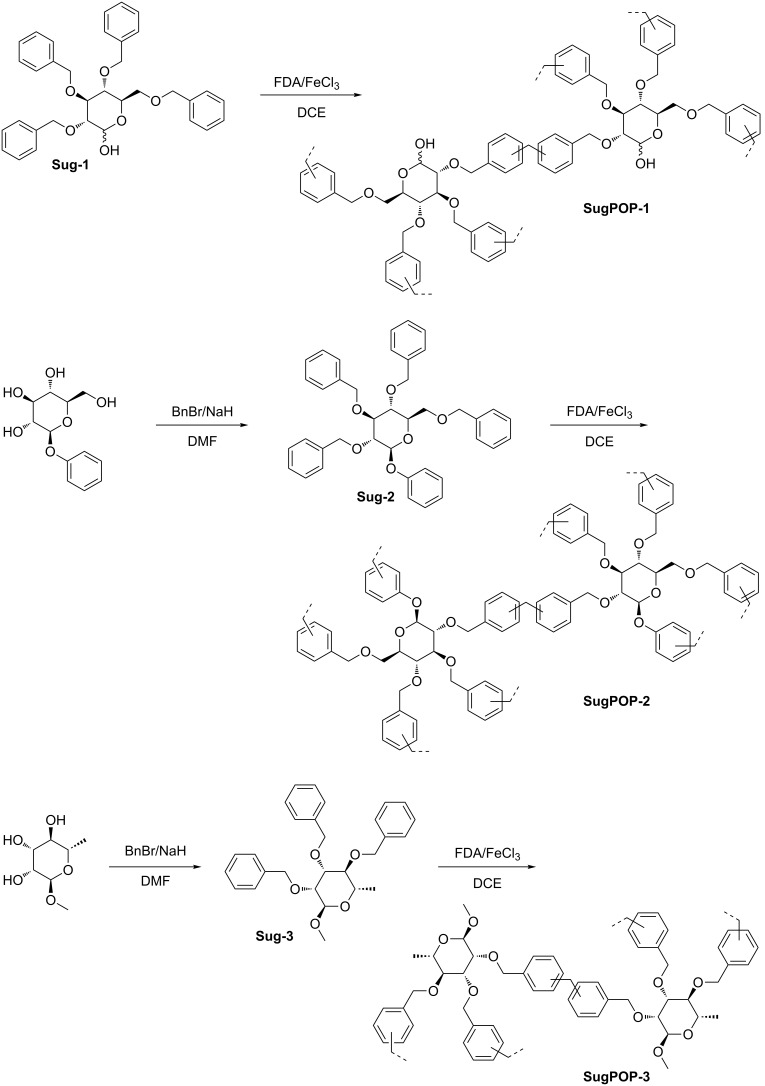
Preparation of polymers **SugPOP-1–3** (FDA: formaldehyde dimethyl acetal).

All the sugar-based porous organic polymers (**SugPOP-1–3**) were synthesized by Friedel–Crafts reaction using FDA as an external cross-linker in a similar way. The preparation routes are shown in Scheme 1. Using benzylated monosaccharides as monomers and FDA as the cross-linker, the Friedel–Crafts cross-linking polymerization is promoted smoothly by anhydrous FeCl_3_ in dry 1,2-dichloroethane (DCE). The monomers were either commercially available (**Sug-1**) or prepared (**Sug-2** and **Sug-3**) by benzylation of free sugars with benzyl bromide and sodium hydride. The chemical structures of **Sug-2** and **Sug-3** have been characterized by ^1^H NMR, ^13^C NMR, and MALDI–TOF MS.

**Figure 1:**
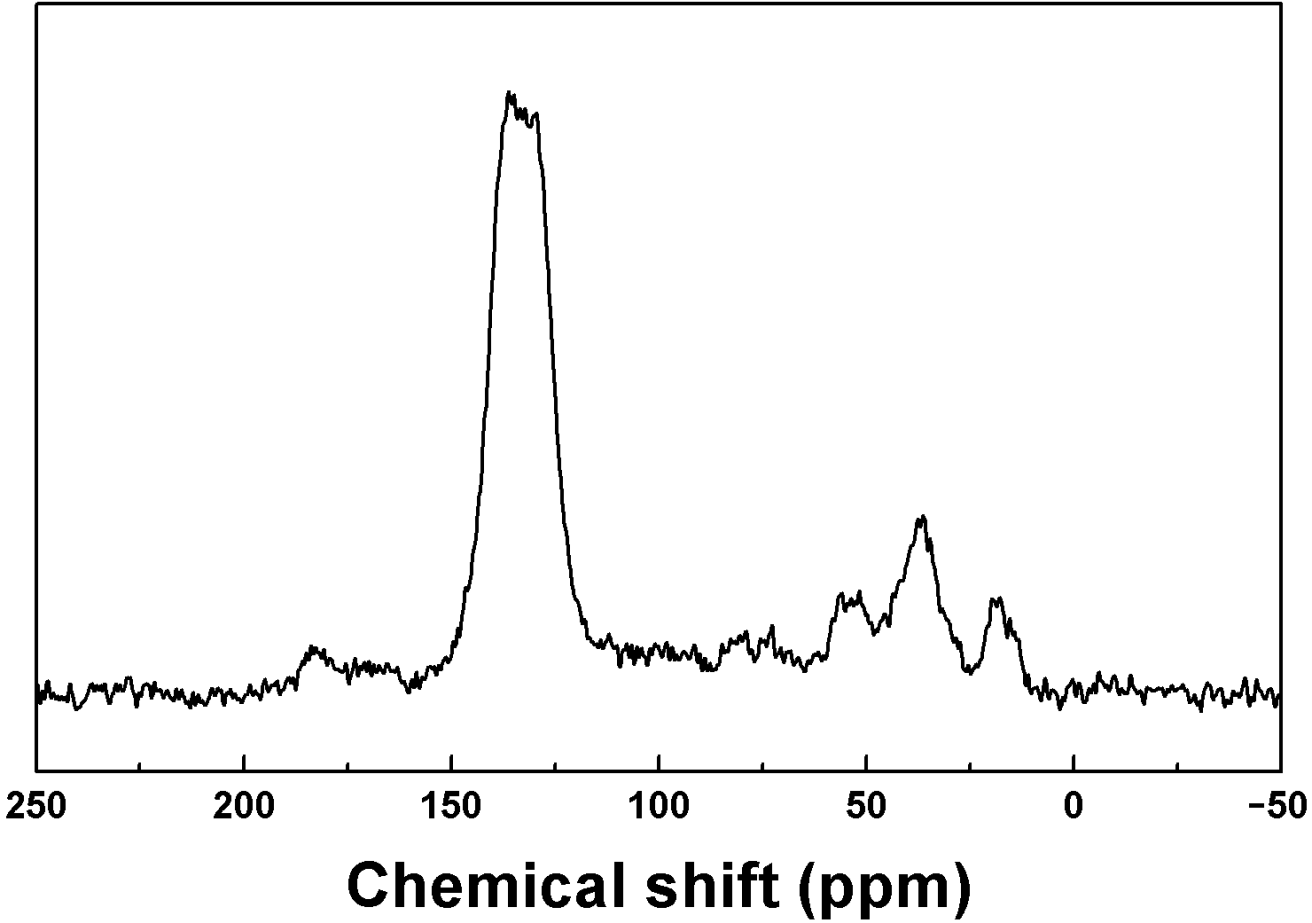
^13^C CP/MAS NMR spectrum of **SugPOP-3**.

The chemical structure of the obtained polymers was confirmed by ^13^C CP/MAS NMR and Fourier transform infrared spectroscopy (FTIR) (Figure S1, Supporting Information File 1). For example, the backbone and structure features of **SugPOP-3** are characterized by ^13^C CP/MAS NMR shown in Figure 1. The resonance signals of the polymer are located at 145–110, 90–50, and 50–15 ppm. The aromatic carbons resonate in the range of 145–110 ppm and the signals at 90–50 ppm are attributed to the carbon backbone of the sugar and methylene carbons connected to the oxygen atom in **SugPOP-3**. Furthermore, the resonance peaks at 50–15 ppm are assigned to the methylene carbons connecting the phenyl rings and to the methyl groups present in the sugar backbone or to the incompletely reacted linker. The ^13^C CP/MAS NMR spectra for **SugPOP-1** and **SugPOP-2** are shown in Figures S2 and S3 (Supporting Information File 1) and show similar resonance intensities to **SugPOP-3**. All of the polymer samples show some common properties of cross-linked polymers such as stability and insolubility in common solvents. The thermal stability of **SugPOP-1–3** was characterized by thermogravimetric analysis (TGA) under nitrogen atmosphere (Figure S4, Supporting Information File 1). The TGA plots show that the decomposition temperature of the polymers is at about 300 °C and there is 35% mass loss when the temperature reaches 800 °C, indicating good thermal stability of the obtained polymers.

**Figure 2:**
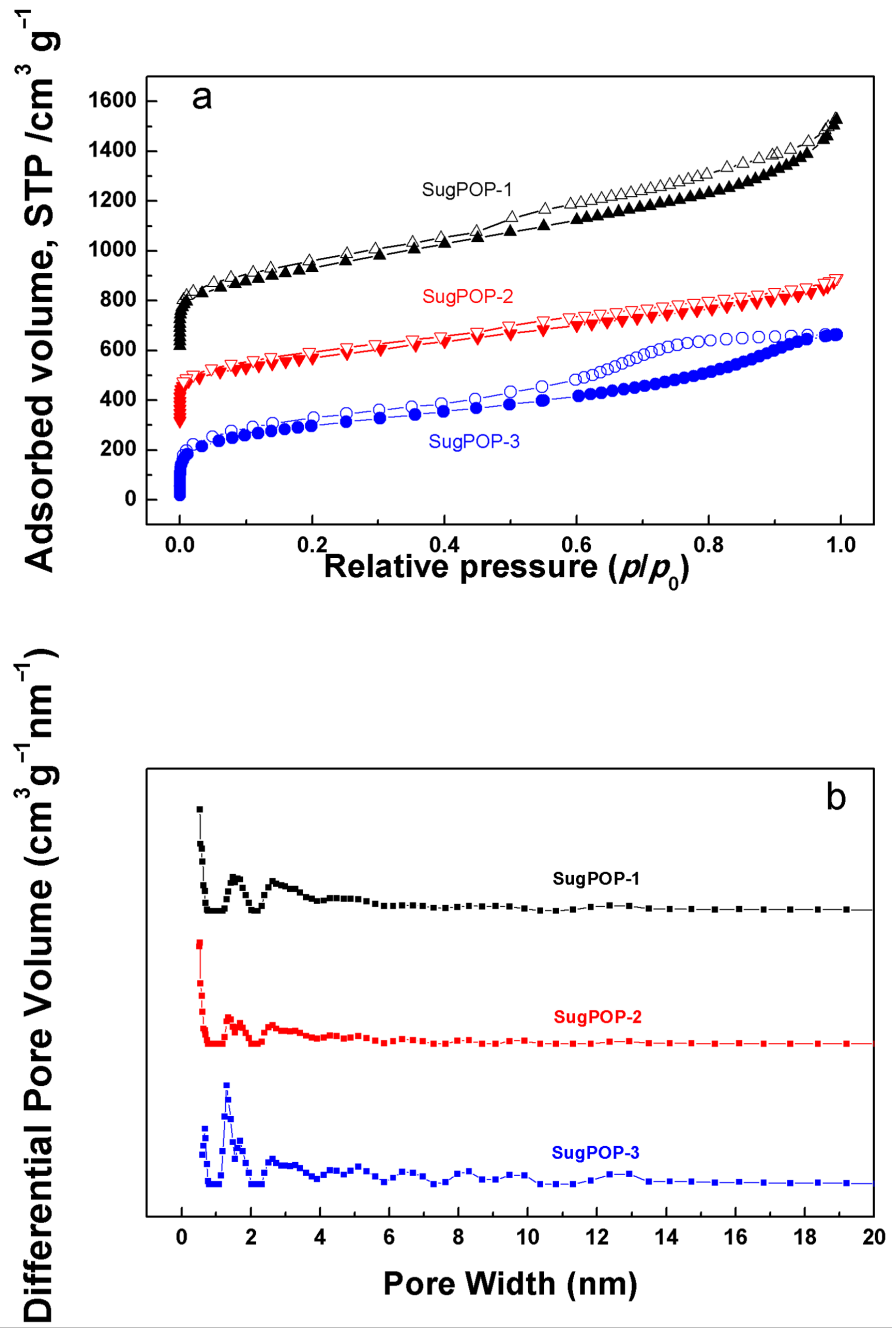
(a) Nitrogen adsorption–desorption isotherms of **SugPOP-1–3** measured at 77 K. For clarity, the isotherms of **SugPOP-1** and **SugPOP-2** were shifted vertically by 600 and 300 cm^3^ g^−1^, respectively. (b) PSD profiles of **SugPOP-1–3** calculated by NLDFT analysis at 77 K.

The nitrogen adsorption–desorption isotherms (Figure 2a) of **SugPOP-1–3** were measured at 77 K to explore the porosity of the obtained polymers. Both **SugPOP-1** and **SugPOP-3** show the combination of type I and IV sorption isotherms according to the IUPAC classification, whereas **SugPOP-2** displays a type I sorption isotherm. A rapid uptake curve reflects the microporous monolayer adsorption tendency at low relative pressure (*p*/*p*_0_ < 0.10). As for **SugPOP-1** and **SugPOP-3**, the significant hysteresis loops (0.50 < *p*/*p*_0_ < 1.00) are consistent with the mesoporous structure. The SSA value (BET) of **SugPOP-1** was found to be as high as 1220 m^2^ g^−1^ and about 1000 m^2^ g^−1^ for **SugPOP-2** and **SugPOP-3**. The PSD profiles for all polymers (based on the nonlinear density functional theory (NLDFT) method) are shown in Figure 2b. The dominant PSD peaks for polymer **SugPOP-2** are located at around 0.53 and 1.35 nm. As for polymers **SugPOP-1** and **SugPOP-3**, their dominant pore size peaks are located around 0.53 and 1.30 nm, associated with mesoporous distribution between 2.2 and 7.0 nm, quantifying their micro/mesoporous features. The main porosity data of the obtained polymers, including SSA, pore volume, and pore size, are calculated based on the corresponding isotherms and listed in Table 1.

**Table 1:** Porosity data and gas sorption performance of **SugPOP-1–3**.

Polymers	*S*_BET_ (m^2^ g^−1^)^a^	*V*_Total_ (cm^3^ g^−1^)^b^	*D*_pore_ (nm)^c^	CO_2_ uptake (wt %)^d^

**SugPOP-1**	1220	1.35	0.53, 1.48, 2.4–6.0	14.4
**SugPOP-2**	970	0.85	0.53, 1.35–2.63	12.8
**SugPOP-3**	1060	0.97	0.68, 1.30, 2.20–7.10	10.5

^a^SSA calculated from the nitrogen adsorption isotherm using the BET method in the relative pressure (*p*/*p*_0_) range from 0.01 to 0.10. ^b^Total pore volume at *p*/*p*_0_ = 0.99. ^c^Pore size calculated from the nitrogen adsorption isotherm using the NLDFT method. ^d^Data were obtained at 1.0 bar and 273 K.

Such HCPs with a high SSA and micro/mesopore distribution inspired us to explore their gas uptake capacity. The CO_2_ adsorption isotherms of the three polymers at 1.0 bar and 273 K are shown in Figure S5 (Supporting Information File 1). The polymer **SugPOP-1** having a higher SSA and pore volume also exhibits a higher CO_2_ adsorption capacity (14.4 wt %) than **SugPOP-2** (12.8 wt %) and **SugPOP-3** (10.5 wt %). Additionally, the hydroxy-group-bearing **SugPOP-1** can form hydrogen bonds with carbon dioxide, which may increase the affinity to carbon dioxide. Compared with the reported polymer Glc-3 [17] (prepared using the same monomer as for **SugPOP-1** and with a similar method), the polymer **SugPOP-1** also possesses a higher CO_2_ adsorption capacity due to its higher porosity.

**Scheme 2:**
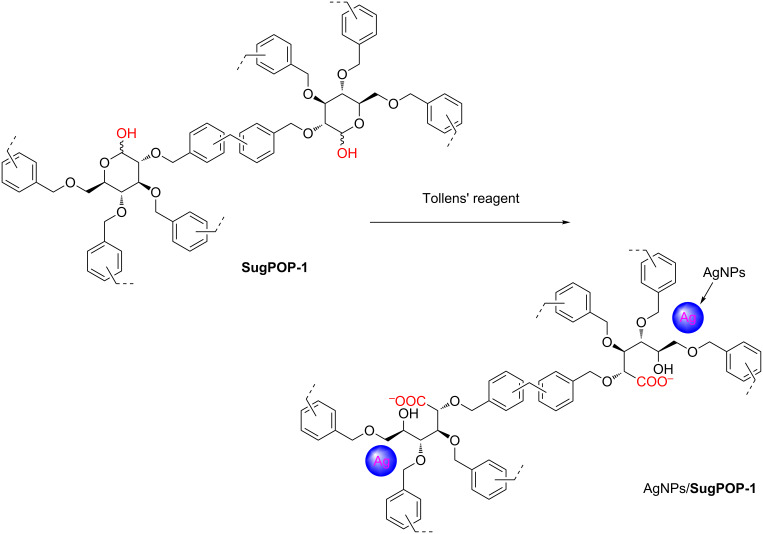
The preparation of AgNPs**/SugPOP-1** composite by the in situ production of AgNPs.

**Figure 3:**
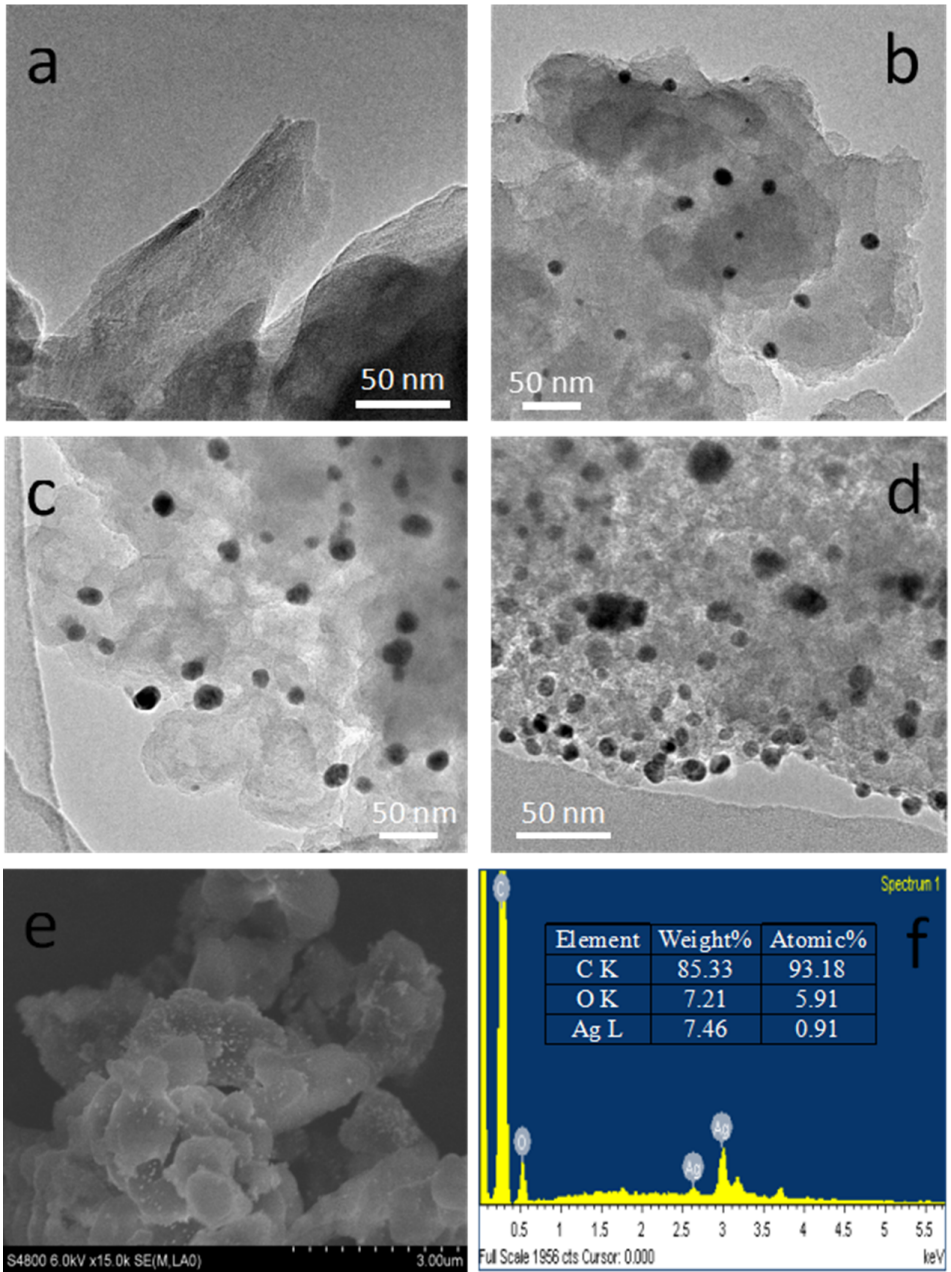
TEM images of the AgNPs**/SugPOP-1** composite taken at different reaction times: (a) 0 h, (b) 8 h; (c) 16 h; (d) 24 h. (e) SEM image and (f) the corresponding EDX data of the AgNPs**/SugPOP-1** composite obtained after reaction for 24 h.

Compared with a class of microporous HCPs obtained by a similar method based on carbohydrates reported by Liu and Dai [17], our obtained porous polymers not only exhibit micro/mesoporous features, but can also be modified and functionalized for further applications. The porous organic polymer **SugPOP-1** containing an aldehyde functionality can be used as the supporting matrix to load AgNPs by treatment with Tollens’ reagent through a redox reaction (Scheme 2) [40]. After **SugPOP-1** was stirred into the freshly prepared Tollens’ reagent solution at 45 °C for 24 h in the dark, the obtained composite was washed with water to remove soluble impurities and dried, resulting in a dark brown solid. The formation process of a AgNPs**/SugPOP-1** composite together with the related morphology of the matrix and AgNPs were studied by TEM. As shown in Figure 3a–d, with increased reaction time, the AgNPs gradually grow and the particle size become apparently larger from 2–10 nm (8 h) to 5–20 nm (24 h). The SEM image of the AgNPs**/SugPOP-1** composite shows that many AgNPs are loaded onto the surface of the matrix (Figure 3e). The corresponding energy-dispersive X-ray spectroscopy (EDX) technique indicates that AgNPs are successfully loaded in **SugPOP-1** (Figure 3f). The weight percentage of carbon, oxygen, and silver is 85.33%, 7.21% and 7.46%, respectively. The atomic percentage of carbon, oxygen and silver is 93.18%, 5.91% and 0.91%, respectively.

The as-synthesized AgNPs**/SugPOP-1** composite was also characterized by X-ray diffraction (XRD) with the results given in Figure S6 (Supporting Information File 1). No crystal diffraction peaks were observed in the **SugPOP-1**, while the diffraction peaks for the AgNPs**/SugPOP-1** composite appeared at 2θ of 38.1°, 44.3°, 64.5°, and 77.4° corresponding to the characteristic peaks of silver [41]. These broad diffraction peaks suggest the formation of small-sized AgNPs. The actual loading capacity of Ag is 5.4 wt % as discerned by TGA under air atmosphere. Meanwhile, the AgNPs**/SugPOP-1** composite exhibits about 2% mass loss at 310 °C and good thermal stability (Figure S7 in Supporting Information File 1).

**Figure 4:**
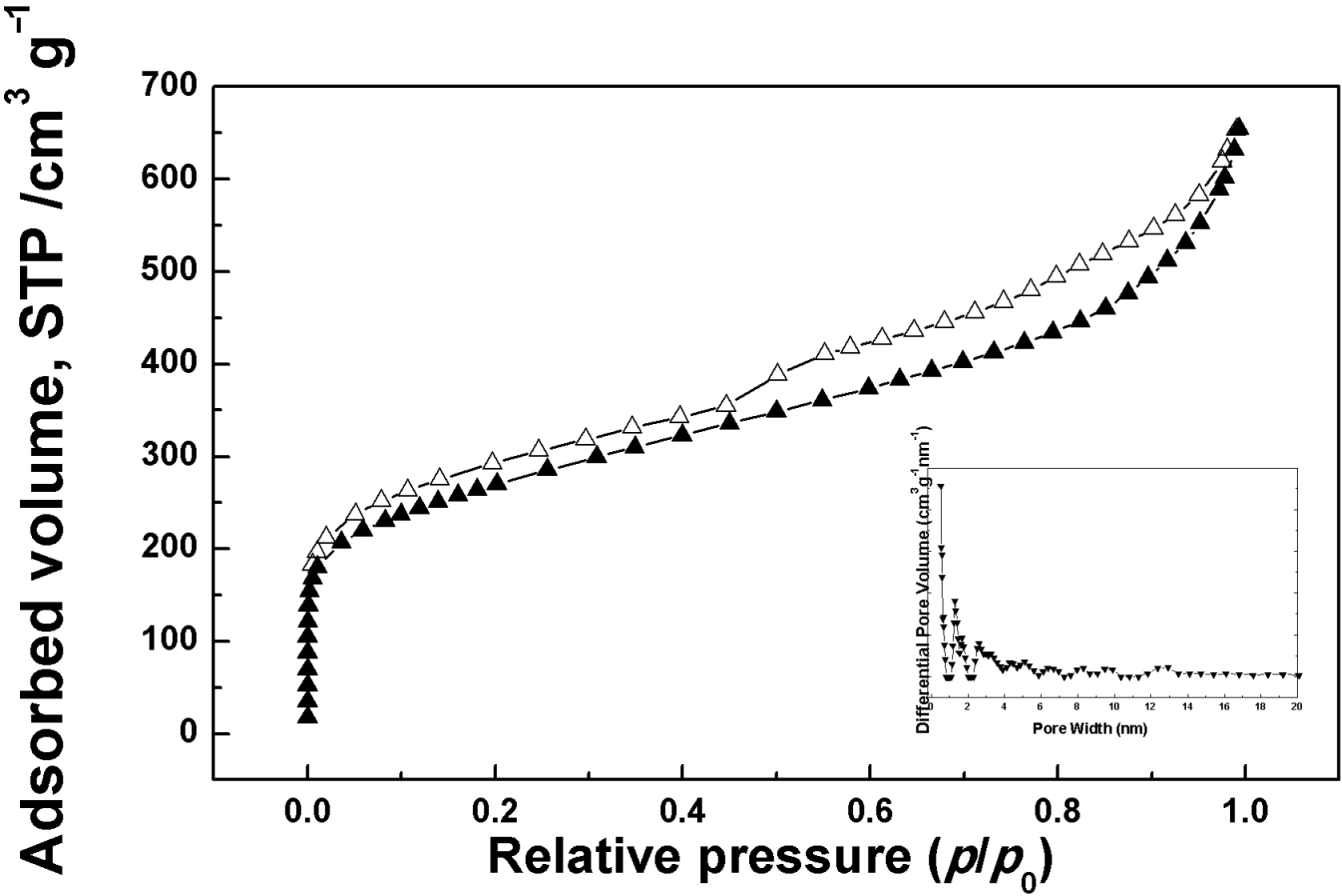
Nitrogen sorption isotherm at 77 K and the pore size distribution profile calculated by NLDFT analysis (inset) of the AgNPs**/SugPOP-1** composite.

The nitrogen adsorption–desorption isotherm of the AgNPs**/SugPOP-1** composite at 77 K and the corresponding PSD profile are shown in Figure 4. The SSA value (BET) of the AgNPs**/SugPOP-1** composite (960 m^2^ g^−1^) is obviously reduced (1220 m^2^ g^−1^ before AgNP loading). However, its nitrogen adsorption–desorption isotherm and PSD are similar to **SugPOP-1**. The as-synthesized AgNPs**/SugPOP-1** composite also exhibits microporous and mesoporous features in which the micropore sizes are between 0.98–1.81 nm and mesopore sizes are in the range of 2–15 nm (based on NLDFT analysis).

The AgNPs loaded on the polymer demonstrate good catalytic activity, which takes on important implications for the conversion of nitro compound precursors or intermediates to the corresponding amino or amine compounds in the preparation of pharmaceuticals and agrochemicals [42–43]. 4-Nitrophenol (4-NP) can cause water pollution, which has aroused widespread concern, while its reduced product, 4-aminophenol (4-AP), is an industrial intermediate for uses such as anticorrosion lubricants and analgesic and antipyretic drugs [44]. The catalytic activity of the AgNPs**/SugPOP-1** composite was tested by the reduction of 4-NP at room temperature with an excess amount of NaBH_4_ as the reducing reagent [45]. In our study, the process of the catalytic reaction was readily followed as the color of the solution turned from yellow to colorless. Both the reactants and products are easily monitored by UV–vis spectroscopy without any formation of appreciable byproduct.

**Figure 5:**
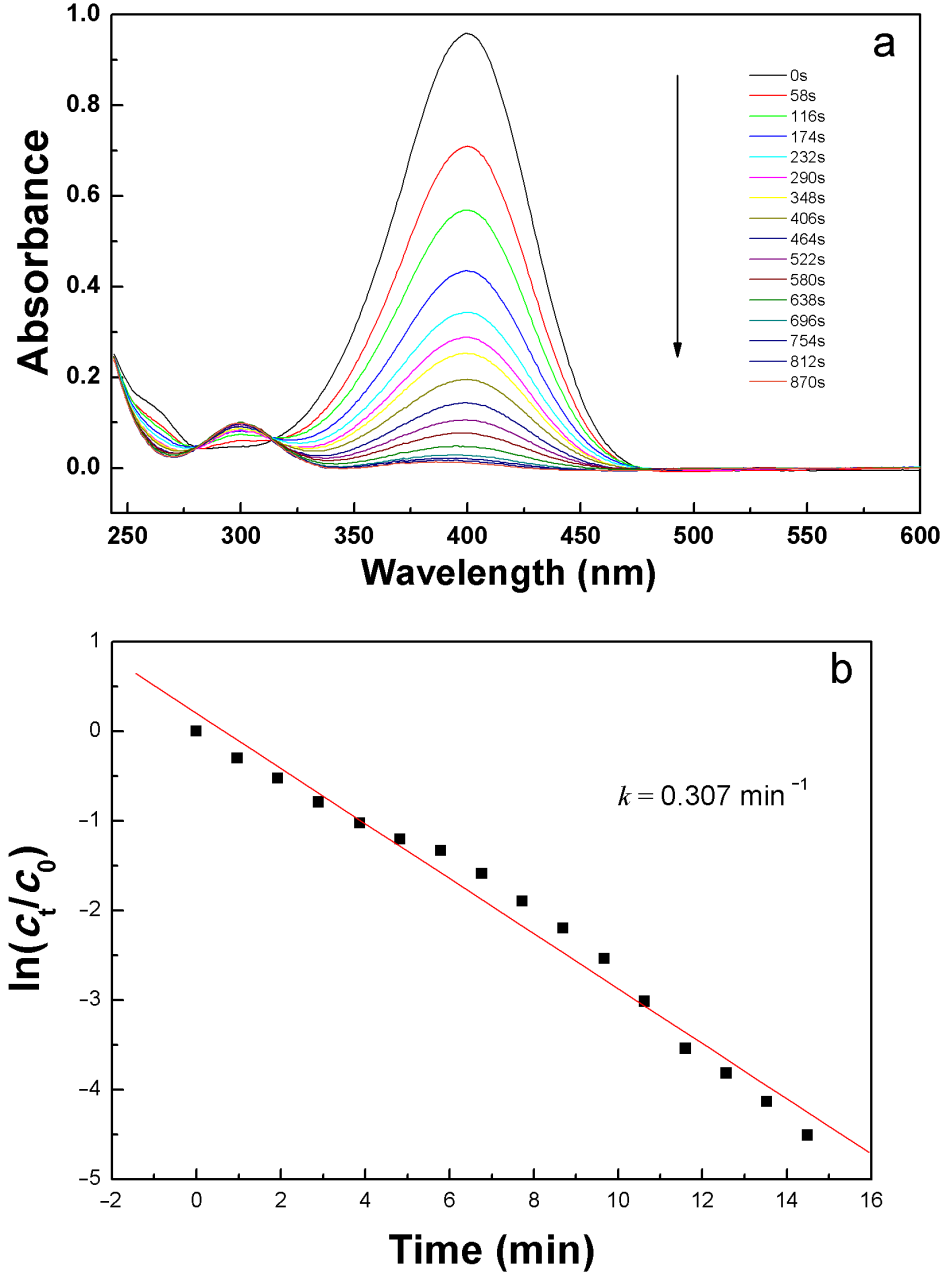
Catalytic performance of the AgNPs**/SugPOP-1** composite. Time-dependent UV–vis spectral changes (a) and the kinetic curve (b) for the catalytic reduction of 4-nitrophenol (4-NP) to 4-aminophenol (4-AP) at room temperature.

Figure 5a shows the performance of the reduction of 4-NP in the presence of the AgNPs**/SugPOP-1** composite as catalyst at different times. As can been seen, the absorption peak at 400 nm gradually decreased, accompanied by emergence of a new peak at approximately 300 nm. Compared to the absorption peak at 317 nm of observed for the neutral 4-NP solution, the absorption at 400 nm is attributed to the 4-nitrophenolate ion. The latter is generated through deprotonation of 4-NP (p*K*_a_ = 7.15) upon the addition of NaBH_4_ [41]. As can be seen from Figure 5a, the absorption peak of the substrate gradually decreased with reaction time due to its conversion. At the same time, the product formation of 4-AP is evident from the new UV–vis band at about 300 nm [46]. There is no byproduct formed during the reaction as the spectra for different reaction times intersect at 283 and 316 nm [47]. After 870 s, the absorption peak at 400 nm disappeared, implying full conversion of 4-NP to 4-AP.

The reaction kinetics of the reduction of 4-NP is considered to be pseudo-first order [48] and can be expressed by the following equation:


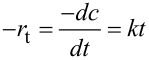


where *r*_t_ is the consumption rate of 4-NP at time *t*, *c*_t_ is the concentration of 4-NP at time *t*, and *k* is the first-order rate constant.

Figure 5b shows the *c*_t_/*c*_0_ and ln(*c*_t_/*c*_0_) changes with time for the reduction of 4-NP in the presence of NaBH_4_ with the AgNPs**/SugPOP-1** composite as catalyst. By the Beer–Lambert law we find that *c*_t_/*c*_0_ is proportional to *A*_t_/*A*_0._ *A*_t_/*A*_0_ was calculated via the corresponding absorbance ratio of the absorption at 400 nm. There is obviously a linear relationship consistent with the pseudo-first-order kinetics between ln(*c*_t_/*c*_0_) and reaction time (*t*). The rate constant *k* of the reaction in the presences of the AgNPs**/SugPOP-1** composite was 0.307 min^−1^ (5.14 × 10^−3^ s^−1^) derived from the slope of the curve in Figure 5b. The active factor *k*_a_ (*k*_a_ = *k*/*m*, *m* is the total mass of catalyst) is considered as a suitable way to judge the activity of the catalyst [49]. As reported, the *k*_a_ of AgNPs/C composite is 1.69 s^−1^ g^−1^ [50], the Fe_3_O_4_@SiO_2_–Ag nanocomposite is 7.76 s^−1^ g^−1^ [51], and the Ag/N–RGO is 7.4 s^–1^ g^–1^ [52]. These are all lower than the value of 51.4 s^–1^ g^–1^ found for the AgNPs**/SugPOP-1** composite prepared in this work. The high catalytic activity is due to the in situ synthesis of AgNPs well-dispersed in the porous polymer support with high SSA, producing more potential catalytic sites, which can promote the interaction between AgNPs and 4-NP to achieve a good catalytic effect.

The catalytic properties of composite materials are closely related to the content and particle size of the AgNPs. If the loading reaction time is short, the content of AgNPs incorporated into the porous polymer is too low and the AgNPs**/SugPOP-1** composite does not exhibit good catalytic activity. Composites with different metal particle sizes will exhibit different catalytic activity. Owing to the smaller particles, possessing more surface atoms available for catalysis, the related catalytic activity of the composite tends to decrease with the increase in the size of the AgNPs. Therefore, the reaction time should not be too long. We found that the optimized loading reaction time was about 24 h.

In the presence of excess BH_4_^−^, the catalytic reduction reaction mediated by the AgNPs**/SugPOP-1** composite could be assumed to follow the monomolecular mechanism [53]. During the reduction process, the interaction between 4-NP and catalytic sites of the AgNPs**/SugPOP-1** composite tend to form adsorbed species and the adsorption behavior to the formation of adsorbed species is described as the Langmuir–Freundlich isotherm [47]. The polymer matrix has higher adsorption capacity for 4-NP due to π–π stacking interactions, which can encourage 4-NP molecules to enter the polymer channel to form the adsorbed species [54]. At the same time, the hydrogen atom is introduced onto the surface of the AgNPs to form Ag–H via BH_4_^−^ reacting with H_2_O. Then, the adsorbed species containing 4-NP react with Ag–H to produce 4-AP [55]. The AgNPs play the role of electron-relaying matter to overcome the kinetic barrier in order to transfer electrons from BH_4_^–^ to 4-NP [56]. In particular, the porous polymers encapsulating AgNPs is thought to accelerate the formation of Ag–H and its reaction with 4-NP. The pore structure of the polymer provides a favorable channel for the entry of 4-NP and the dissociation of 4-AP. The AgNPs embedded in the porous polymer remain active and the activity remains unaltered during the whole process [18].

## Conclusion

The preparation of hypercross-linked polymers based on perbenzylated monosugars by Friedel–Crafts reaction using FDA as an external cross-linker is reported. Considering that the features of the polyhydroxylated structures derived from the monosaccharides have an effect on the SSA and PSD of the prepared porous polymers, porosity tuning could be achieved with three monomers with different molecular structures. The obtained polymers exhibit mainly microporous and mesoporous features with an SSA (BET) of about 1000 m^2^ g^−1^. As for one of the obtained porous polymers containing a hemiacetal glucose motif (**SugPOP-1**), AgNPs were smoothly embedded into the material by chemical reduction of freshly prepared Tollens’ reagent, allowing in situ formation of AgNPs in the polymer matrix. With a high porosity and micro-/mesoporous features, the AgNP-loaded polymer composite, AgNPs**/SugPOP-1**, exhibited good catalytic activity in the reduction of 4-NP at room temperature with a high activity factor (51.4 s^−1^ g^−1^). This reflects the high catalytic activity of AgNPs**/SugPOP-1** with micro-/mesoporous features and implies important applications of nitro compound precursors for the preparation of pharmaceuticals and agrochemicals.

## Experimental

### Synthesis of perbenzyl phenyl β-D-glucopyranoside (**Sug-2**)

Sodium hydride (60%, 0.43 g, 10.85 mmol) was added portionwise to a solution of phenyl β-D-glucopyranoside (93 mg, 0.36 mmol) in DMF (5.0 mL) over 40 min under nitrogen atmosphere in an ice bath. After being stirred at room temperature, benzyl bromide (0.4 mL, 3.37 mmol) was added to the mixture. The resulting mixture was stirred for 4 h at room temperature and then ice water was added to quench the reaction. The suspension was extracted with ethyl acetate (2 × 50 mL). The combined organic layer was washed with water (3 × 50 mL) and dried with anhydrous sodium sulfate. After removing the solvent under reduced pressure, the residue was chromatographed on silica gel to give **Sug-2** as a white solid (149 mg, 67%). ^1^H NMR (400 MHz, CDCl_3_) δ (ppm) 7.31 (s, 20H), 7.20 (s, 2H), 7.13–6.97 (m, 3H), 5.12–4.90 (m, 3H), 4.84 (t, *J* = 11.1 Hz, 3H), 4.65–4.48 (m, 3H), 3.73 (m, 6H); ^13^C NMR (100 MHz, CDCl_3_) δ (ppm) 157.4, 138.6, 138.3, 138.2, 138.1, 129.6, 128.5, 128.4, 128.3, 128.0, 127.9, 127.8, 127.7, 127.6, 122.7, 116.9, 101.7, 84.7, 82.1, 77.8, 75.8, 75.2, 75.1, 73.5, 68.9; MS (MALDI–TOF) *m*/*z*: [M + Na] calcd for C_40_H_40_O_6_, 639.3; found: 639.4.

### Synthesis of perbenzyl methyl α-L-rhamnopyranoside (**Sug-3**)

Sodium hydride (160 mg, 60%, 6.72 mmol) was added to a solution of methyl α-L-rhamnopyranoside (200 mg, 1.12 mmol) in DMF (5.0 mL) in an ice bath over 40 min under a nitrogen atmosphere. Then benzyl bromide (470 μL, 3.93 mmol) was added and the reaction mixture was stirred at room temperature for 4 h. After completion (TLC and carbonation), the organic layer was extracted twice with ethyl acetate (50 mL). The combined organic layer was washed three times with water and dried with anhydrous sodium sulfate. The solvent was removed under reduced pressure and the residue was purified by silica gel column chromatography to give a colorless solid foam (286 mg, 57%). ^1^H NMR (400 MHz, CDCl_3_) δ (ppm) 7.38–7.19 (m, 15H), 4.92 (d, *J* = 10.9 Hz, 1H), 4.71 (q, *J* = 12.7 Hz, 2H), 4.66–4.52 (m, 4H), 3.87–3.70 (m, 2H), 3.61 (dd, *J* = 18.9, 9.9 Hz, 2H), 3.26 (s, 3H), 1.38–1.26 (m, 3H); ^13^C NMR (100 MHz, CDCl_3_) δ (ppm) 138.8, 138.7, 138.5, 128.5, 128.1, 128.0, 127.8, 127.7, 127.6, 99.2, 80.6, 80.3, 75.5, 74.9, 72.9, 72.2, 68.0, 54.7, 18.1; MS (MALDI–TOF) *m*/*z*: [M + Na] calcd for C_28_H_32_O_5_, 471.2; found: 471.2.

### Synthesis of polymers **SugPOP-1–3**

The representative synthesis procedure was as follows (**SugPOP-1**). Anhydrous FeCl_3_ (180 mg, 1.11 mmol) was added to a stirred solution of **Sug-1** (100 mg, 0.18 mmol) and FDA (99 μL, 1.11 mmol) in 10 mL dry DCE under a nitrogen atmosphere. After the solution was well mixed, the resulting mixture was heated to 45 °C for 5 h and 85 °C for 19 h. The obtained precipitate was washed three times with methanol and THF, respectively. The residue was further purified by Soxhlet extraction with methanol and THF for 24 h each, then dried under reduced pressure at 50 °C for 24 h to give **Sug-1** as a brown powder (96 mg, 89%).

Following the same procedure as described for **SugPOP-1**, **SugPOP-2**, and **SugPOP-3** were prepared from **Sug-2** and **Sug-3**, respectively, with yields of about 90%.

### Preparation of AgNPs/**SugPOP-1** composite

**SugPOP-1** (101 mg) was added to a freshly prepared Tollens’ reagent solution (15 mL). The reaction mixture was stirred at 45 °C in the dark for 24 h. The obtained product was filtered and washed with water and ethanol and then dried under reduced pressure at 45 °C for 24 h to give AgNPs**/SugPOP-1** composite as a brown solid (100 mg).

### Catalytic reduction of 4-nitrophenol (4-NP) by AgNPs/**SugPOP-1** composite

To investigate the catalytic performance of the AgNPs**/SugPOP-1** composite, the reduction of 4-NP was performed in a quartz cuvette (1 cm optical path, 4 mL volume) in the presence of sodium borohydride (NaBH_4_). 4-NP (1.44 mM, 0.10 mL) and a freshly prepared aqueous NaBH_4_ solution (6.87 mM, 2.80 mL) were added in the quartz cuvette. Then, the AgNPs**/SugPOP-1** composite (1.0 mg/mL, 0.10 mL) suspended in deionized water was subsequently added to the above solution and the reaction progress monitored by UV–vis spectroscopy. The absorption spectra were measured at room temperature by recording absorbance from 244–600 nm within defined time intervals. The reduction reaction was conducted within minutes after the solution was prepared to minimize decomposition of NaBH_4_.

## Supporting Information

File 1 Additional spectra.
IR spectra of the **SugPOP-1–3**; ^13^C CP/MAS NMR spectra of **SugPOP-1** and **SugPOP-2**; TGA curves of polymers **SugPOP-1–3** under nitrogen atmosphere; CO_2_ adsorption isotherms of **SugPOP-1–3** with pressure up to 1.13 bar; X-ray diffraction patterns of **SugPOP-1** and AgNPs**/SugPOP-1** composite; TGA plot of the AgNPs**/SugPOP-1** composite under air atmosphere; ^1^H NMR, ^13^C NMR, and MS spectra of new monomers are provided.
