# Supplementary material for: Sugar-based micro/mesoporous hypercross-linked polymers with in situ embedded silver nanoparticles for catalytic reduction
Source: Beilstein J Org Chem. 2017 Jun 22;13:1212–21. doi: 10.3762/bjoc.13.120 (PMC5496582; doi:10.3762/bjoc.13.120)
Supplement: File 1 — Additional spectra. IR spectra of the SugPOP-1–3; 13C CP/MAS NMR spectra of SugPOP-1 and SugPOP-2; TGA curves of polymers SugPOP-1–3 under nitrogen atmosphere; CO2 adsorption isotherms of SugPOP-1–3 with pressure up to 1.13 bar; X-ray diffraction patterns of SugPOP-1 and AgNPs/SugPOP-1 composite; TGA plot of the AgNPs/SugPOP-1 composite under air atmosphere; 1H NMR, 13C NMR, and MS spectra of new monomers are provided. [file Beilstein_J_Org_Chem-13-1212-s001.pdf]

Supporting Information
for
Sugar-based micro/mesoporous hypercross-linked polymers with in situ embedded silver nanoparticles for catalytic reduction

Qing Yin[1,2], Qi Chen [2*§], Li-Can Lu[1*$] and Bao-Hang Han[2*¶]

Address: [1]College of Chemistry, Xiangtan University, Xiangtan 411105, China and [2]CAS Key Laboratory of Nanosystem and Hierarchical Fabrication, CAS Center for Excellence in Nanoscience, National Center for Nanoscience and Technology, Beijing 100190, China

Email: Qi Chen - chenq@nanoctr.cn; Li-Can Lu - lulican@xtu.edu.cn; Bao-Hang Han - hanbh@nanoctr.cn

*Corresponding author

[§]Tel: +86 10 8254 5708

[$]Tel: +86 731 5829 2207

[¶]Tel: +86 10 8254 5576

Materials

Formaldehyde dimethyl acetal (FDA) was purchased from Aldrich Chemicals. Phenyl β-D-glucopyranoside was purchased from J&K Scientific Ltd. Methyl α-L-rhamnopyranoside with a purity of 98% was purchased from Acros Ltd. 2,3,4,6-Tetra-*O*-benzyl-D-glucopyranose (**Sug-1**) was purchased from Sahn Shanghai Chemical Technology Co. Ltd. Other chemicals and reagents were purchased from commercial chemical suppliers and used without further purification unless otherwise stated. *N*,*N*-Dimethylformamide (DMF), 1,2-dichloroethane (DCE), and tetrahydrofuran (THF) were dried before use.

Instrumental characterization

^{1}H NMR and ^{13}C CP/MAS NMR spectra were recorded with a Bruker Advance III 400 NMR spectrometer (Bruker, Germany). Mass spectra (MS) were obtained using a Microex LRF MALDI-TOF mass spectrometer (Bruker Daltonics, U.S.A). Infrared (IR) spectra were collected with a Spectrum One Fourier transform infrared (FTIR) spectrometer (PerkinElmer Instruments Co. Ltd., U.S.A.) as KBr pellets. The sample was mixed with KBR followed by compression to obtain suitable disks and 16 scans were signal-averaged. Scanning electron microscopy (SEM) observations were carried out using S-4800 microscope (Hitachi Ltd., Japan) equipped with a Horiba energy dispersive X-ray (EDX) at an accelerating voltage of 6.0 kV. Transmission electron microscopy (TEM) observations were obtained using a Tecnai G^{2} S-TWIN

microscope (FEI, U.S.A.) at an accelerating voltage of 200 kV. X-ray diffraction (XRD) patterns were recorded from 10° to 90° by a Philips X'Pert PRO X-ray diffraction instrument (Philips, Netherlands). Thermogravimetric analysis (TGA) was conducted on a Pyris Diamond thermogravimetric/differential thermal analyzer (PerkinElmer Instruments Co. Ltd., U.S.A.) by heating the samples at 10 °C min^{-1} to 800 °C under nitrogen/air atmosphere. CO_2 uptake was obtained using Micromeritics TriStar II 3020 (Micromeritics, U.S.A.). Nitrogen sorption isotherms were obtained using 3-Flex accelerated surface area and porosity analyzers (Micromeritics, U.S.A.). The samples were degassed at 120 °C for 12 h and in situ at 120 °C for another 10 h. The obtained adsorption–desorption isotherms were evaluated to give the porosity parameters, including Brunauer–Emmett–Teller (BET) specific surface area, pore size, and pore volume.

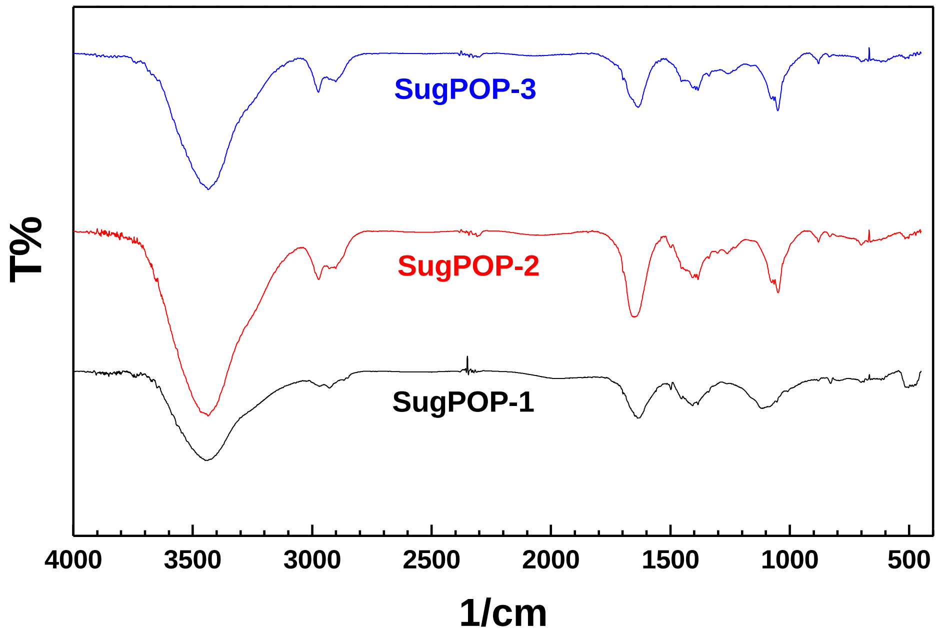

Figure S1: IR spectra of polymers **SugPOP-1–3**.

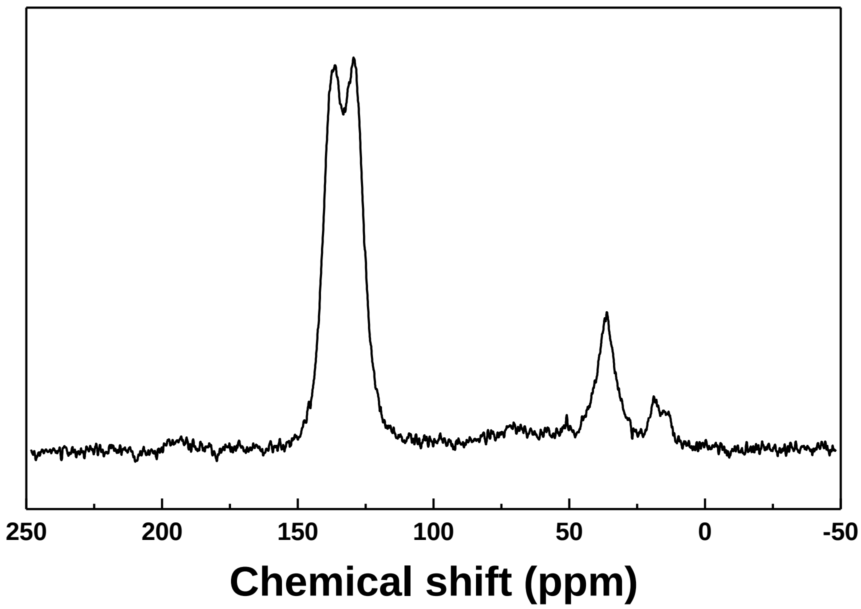

Figure S2: ^{13}C CP/MAS NMR spectrum of **SugPOP-1**.

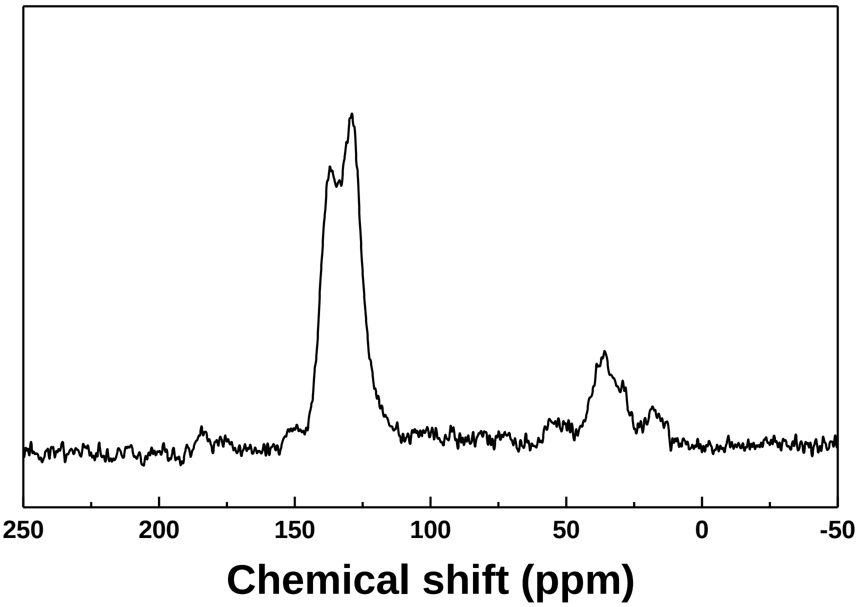

Figure S3: ^{13}C CP/MAS NMR spectrum of **SugPOP-2**.

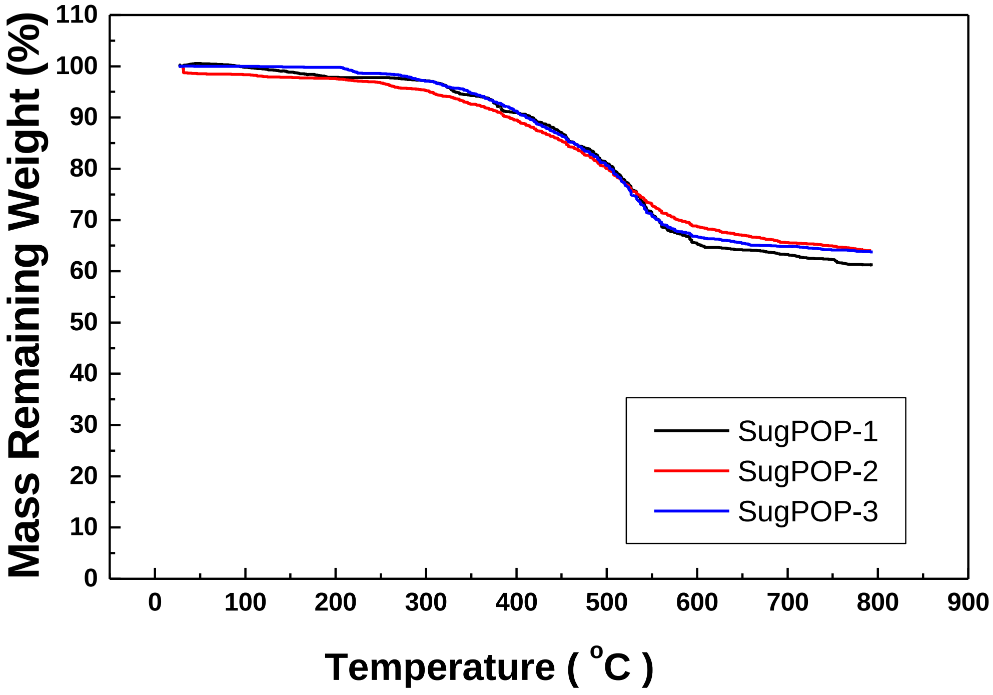

Figure S4: TGA curves of polymers **SugPOP-1–3** under nitrogen atmosphere.

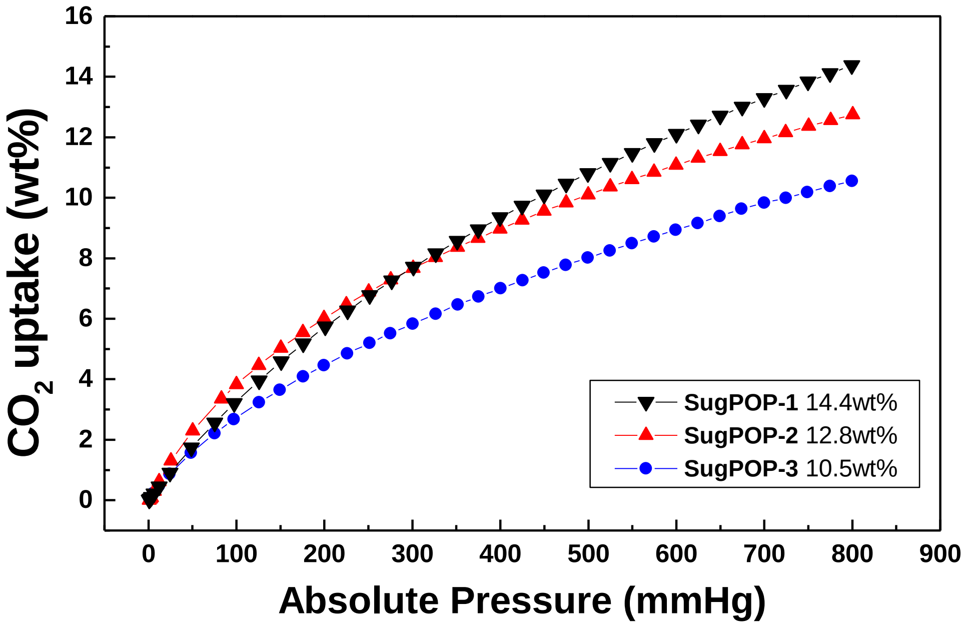

Figure S5: CO_2 adsorption isotherms of polymers **SugPOP-1–3** at 273 K.

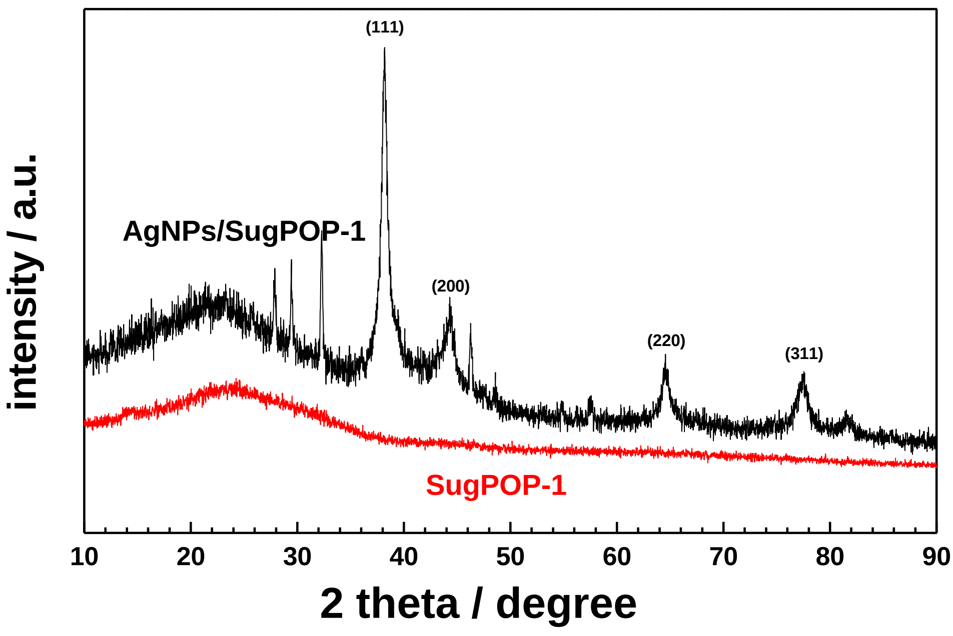

Figure S6: X-ray diffraction patterns of **SugPOP-1** and AgNPs/**SugPOP-1** composite.

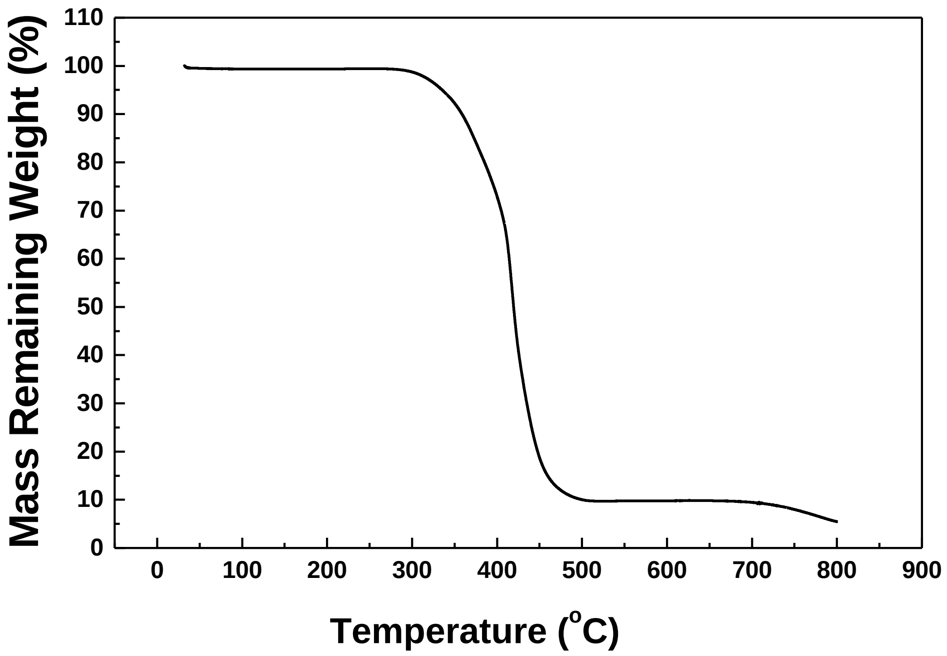

Figure S7: TGA plot of the AgNPs/**SugPOP-1** composite under air atmosphere.

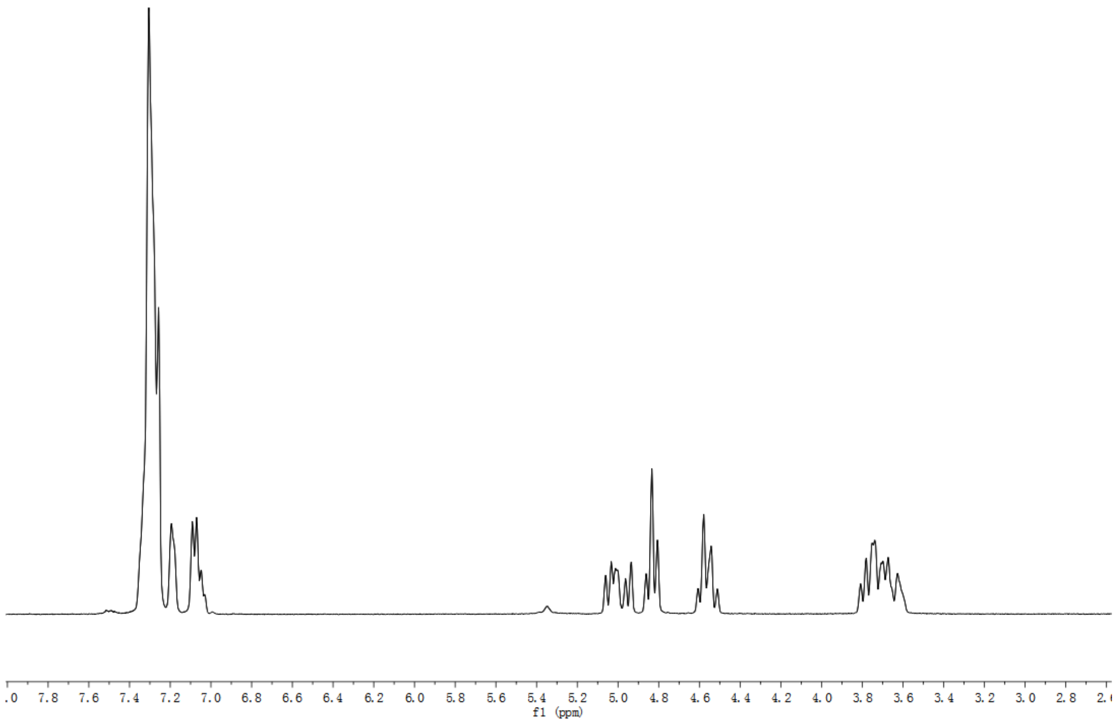

^{1}H NMR (400 MHz, $CDCl_3$) spectrum of **Sug-2**

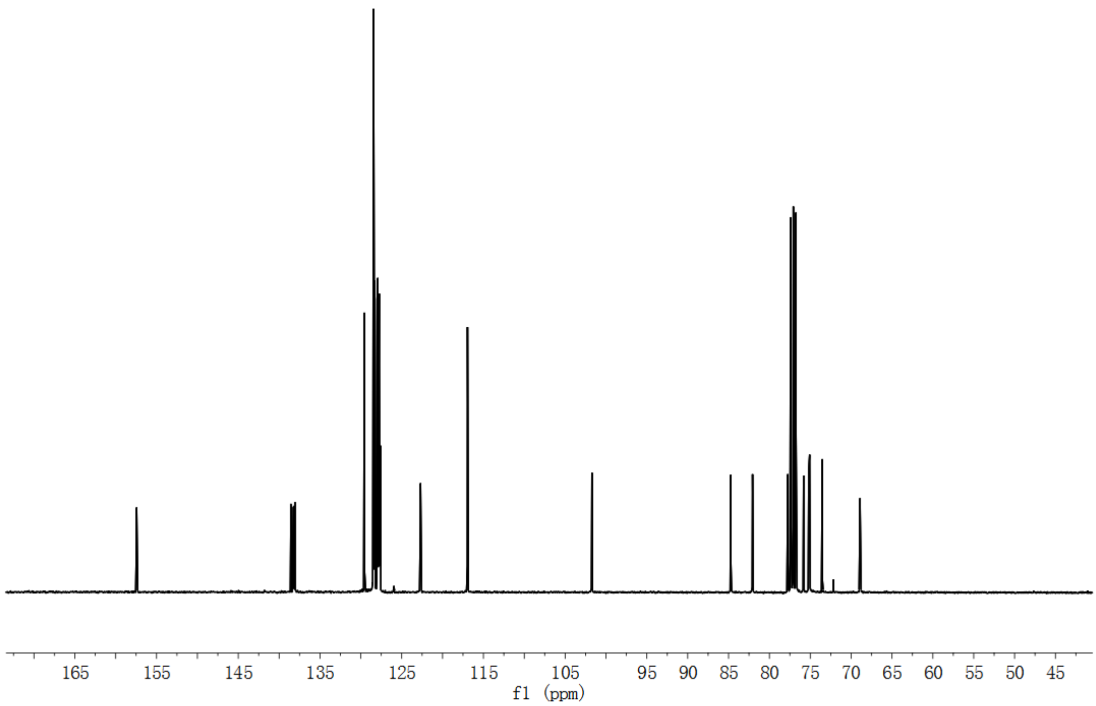

^{13}C NMR (100 MHz, $CDCl_3$) spectrum of **Sug-2**

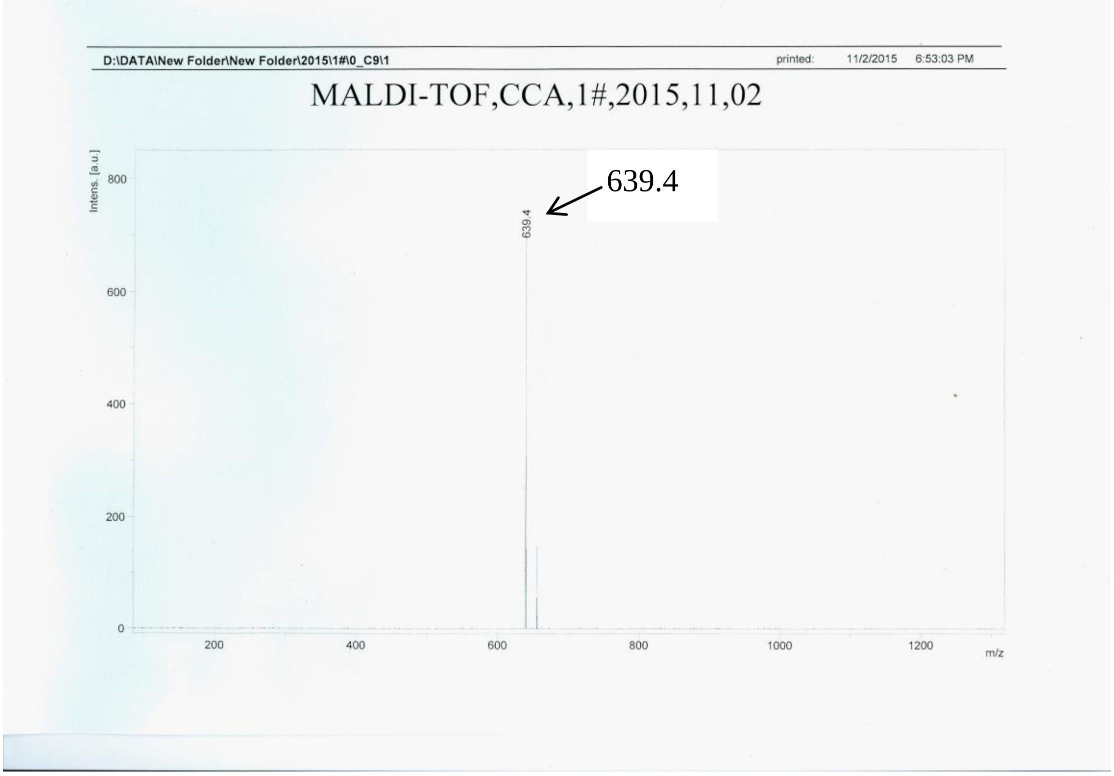

MS of **Sug-2**

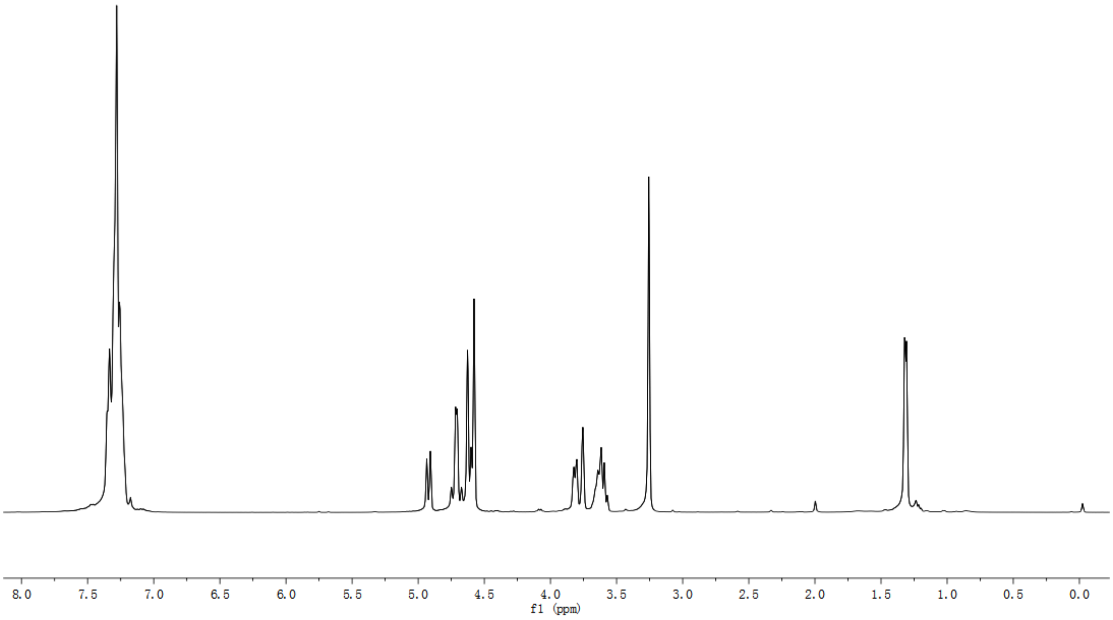

^{1}H NMR (400 MHz, $CDCl_3$) spectrum of **Sug-3**

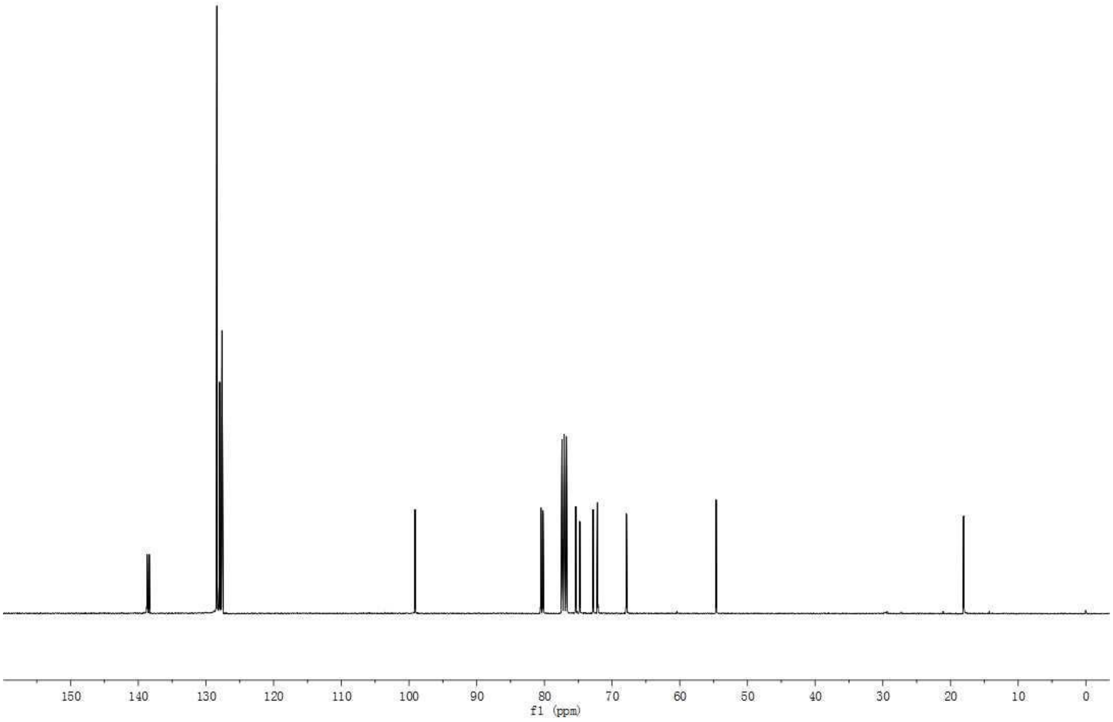

^{13}C NMR (100 MHz, $CDCl_3$) spectrum of **Sug-3**

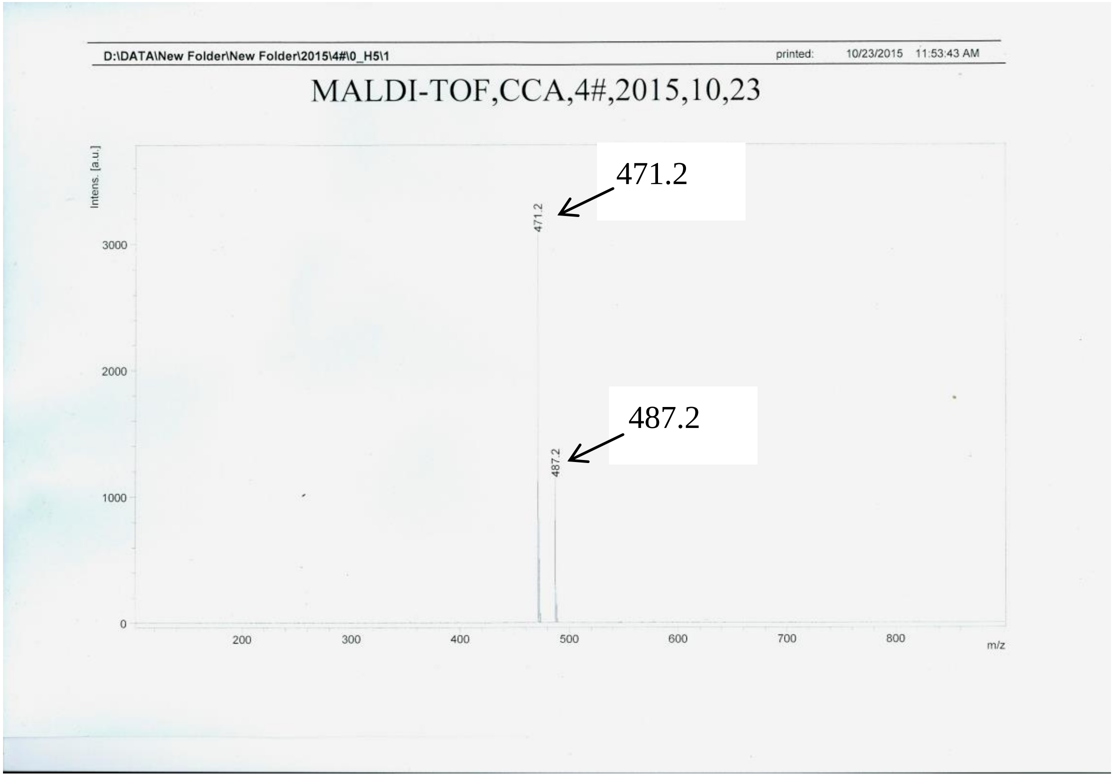

MS of **Sug-3**
